# The Relationship between Planar and SPECT/CT Parameters and Functional Markers in Primary Hyperparathyroidism

**DOI:** 10.3390/diagnostics13203182

**Published:** 2023-10-11

**Authors:** Guler Silov, Serpil Erdogan Ozbodur

**Affiliations:** 1 Department of Nuclear Medicine, Samsun University Faculty of Medicine, Samsun 55200, Turkey; 2 Department of Nuclear Medicine, Gazi State Hospital, Samsun Provincial Health Directorate, Samsun 55070, Turkey; serpilerdogan2002@yahoo.com

**Keywords:** primary hyperparathyroidism, dual-phase parathyroid scintigraphy, SPECT/CT, quantitative analysis, parathyroid adenoma volume

## Abstract

This study aimed to investigate the relationship between quantitative and volumetric parameters of technetium-99-methoxyisobutylisonitrile (99mTc-MIBI) single-photon emission computed tomography/computed tomography (SPECT/CT) imaging and hormonal and biochemical markers in primary hyperparathyroidism (PHPT) patients with single adenoma. In this retrospective study, 70 patients with a single adenoma who underwent 99mTc-MIBI imaging for the diagnosis of PHPT were examined. Early and delayed MIBI lesion/background ratios (eLBR and dLBR), early and delayed lesion/thyroid ratio (eLTR and dLTR), and retention index (RI) were calculated as planar dual-phase scintigraphy parameters. Adenoma volume (Svol) and parathormone (PTH)/Svol ratio were measured as SPECT/CT-derived parameters. Calcium levels exhibited a positive correlation with eLBR (r = 0.33, *p* < 0.001), dLBR (r = 0.29, *p* = 0.01), dLTR (r = 0.31, *p* < 0.001), and PTH/Svol (r = 0.38, *p* < 0.001). PTH did not correlate with planar parameters and Svol. Among the imaging parameters, only the PTH/Svol ratio showed a negative correlation with phosphorus levels (r = −0.29, *p* = 0.02). For predicting disease severity, the PTH/Svol ratio exhibited similar diagnostic performance to PTH and phosphorus levels but outperformed the eLBR and dLBR. Both planar and SPECT-derived parameters can provide valuable insights into the functional status of the parathyroid adenoma and the associated disease severity. PTH/Svol ratio, combining imaging and laboratory findings to provide a more comprehensive approach to patient care, could be an exciting new indicator.

## 1. Introduction

Primary hyperparathyroidism (PHPT) is the most common form of hyperparathyroidism (HPT), characterized by the excessive production of parathormone (PTH) by the parathyroid glands, and frequently leads to hypercalcemia [1]. Usually, PHPT is the result of a single parathyroid adenoma (80%), although it can also come about from multiple adenomas, parathyroid hyperplasia, or rarely, parathyroid carcinoma (<1%). Multigland disorder (MGD), characterized by multiple adenomas or hyperplasia of multiple glands, is encountered in 15–20% of those with PHPT [2]. Therefore, accurately determining the location of abnormal parathyroid glands using imaging methods plays a crucial role in surgical planning and success [3].

The technetium-99m methoxyisobutylisonitrile (99mTc-MIBI) dual-phase scintigraphy (DPS) is retained longer in parathyroid adenomas compared to healthy thyroid and parathyroid tissues [4,5]. Therefore, it is commonly used in clinical practice to determine the localization of parathyroid adenoma. However, serum calcium levels reflecting the severity of PHPT might induce variations in 99mTc-MIBI kinetics by affecting the membrane potential [6]. Few studies have indicated a positive correlation between serum calcium and PTH levels and adenoma volume in patients with parathyroid adenomas [7,8,9,10]. On the other hand, we have not encountered a study that evaluates the relationship between quantitative and volumetric parameters of 99mTc-MIBI and the hormonal and biochemical markers of PHPT.

We hypothesized that there might be a positive relationship between the functional status of PHPT and the quantitative and volumetric parameters of 99mTc-MIBI. Therefore, this study aimed to investigate the relationship between quantitative and volumetric parameters of 99mTc-MIBI imaging and hormonal and biochemical markers in PHPT patients with single adenoma.

## 2. Materials and Methods

Following the principles set forth in the Declaration of Helsinki, this retrospective study was conducted at the Samsun Gazi State Hospital Nuclear Medicine Department from January 2019 and March 2023. The study received approval from the local ethics committee (Approval Date: April 2023, Decision No. 2023000119).

### 2.1. Study Population

A total of 79 patients who underwent 99mTc-MIBI DPS imaging for the diagnosis of PHPT during the study period were retrospectively examined. Inclusion criteria encompassed patients exhibiting a solitary adenoma, corroborated by biochemical, pathological, and imaging indications of HPT, and without a history of using calcium-decreasing medications. Exclusion criteria were documented multinodular goiter (MNG) (*n* = 3), secondary hyperparathyroidism (*n* = 4), and MGD (*n* = 2). After the exclusion process, 70 patients (mean age: 56.2 ± 13.1; range: 24–84 years), including 54 women and 16 men, exhibiting evidence of PHPT, with a single adenoma and a positive 99mTc-MIBI test, were included in the analysis.

According to the current guideline, the calcium level defined for parathyroidectomy criteria in non-symptomatic PHPT is 1.0 mg/dL higher than the maximum limit of normal [11]. Accordingly, patients were divided into two categories based on the severity of disease: mild (with calcium levels ranging from 10.5 to 11.5 mg/dL; *n* = 50) and marked hypercalcemia (with calcium levels exceeding 11.5 mg/dL; *n* = 20) [9,12].

### 2.2. Study Protocol

The demographic, clinical, and imaging data were extracted from the electronic records of the patients.

Although dual-phase parathyroid scintigraphy is a time-consuming procedure for nuclear medicine practitioners and patients, it is recognized in clinical practice that most parathyroid adenomas retain their activity and become more visible in delayed images [13]. Therefore, all patients underwent early and late 99mTc-MIBI DPS. All patients were receptive to the Dual-Time-Point (DTP) protocol. They were informed in advance about the 90-min. duration for the delayed scan, and their feedback indicated that the extended study was generally well-tolerated. Patients were repositioned on the second scan. Although one-to-one image matching is not required for quantitative analysis in both early and delayed imaging, consistent positioning was achieved in the subjects for both scans. In the SPECT/CT phase, no mismatches were observed as the patient was fixed on the table during both SPECT and CT acquisition. In 29 cases where a single adenoma was visually identified in early and delayed planar scintigraphy, single-photon emission computed tomography/computed tomography (SPECT/CT) was not performed to avoid the unnecessary risk of additional radiation exposure. For the 41 cases where an adenoma was not distinctly identified in both early and delayed planar scintigraphy, SPECT/CT imaging was conducted. The early and delayed planar 99mTc-MIBI DPS images and reconstructed cross-sectional SPECT/CT images were viewed and analyzed by consensus between two nuclear medicine physicians.

### 2.3. Parathyroid Imaging

**Figure 1:**
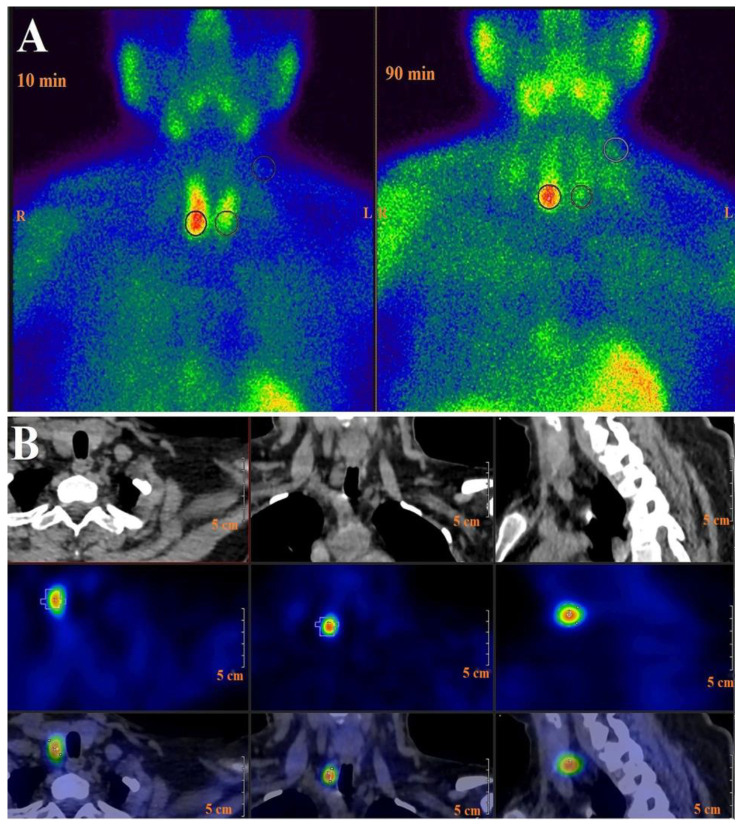
(**A**) Calculation of early lesion to background ratio (eLBR), early lesion to thyroid ratio (eLTR); delayed lesion to background ratio (dLBR) and delayed lesion to thyroid ratio (dLTR) from the region of interest (ROI). (**B**) Determination of parathyroid adenoma volume (Svol) from the SPECT imaging using semi-automated volume of interest option.

Based on a guideline outlining comprehensive techniques for parathyroid imaging, both 99mTc-MIBI DPS and SPECT/CT were utilized [13]. 99mTc-MIBI DPS was performed immediately after intravenous injection of a dose of 600–900 MBq. Early (10 min after injection) and delayed (90 min) planar images were acquired using a SPECT/CT gamma camera. SPECT/CT acquisitions were made after the delayed planar images with the patient in the same position with 64 projections over 180° in a circular orbit. The acquisition time per step was 30 s. SPECT images were reconstructed on 128 × 128 matrices using a Butterworth pre-processing filter and an iterative algorithm (ordered-subset expectation maximization). After SPECT acquisition, a spiral CT scan was performed for anatomical localization and attenuation correction without contrast enhancement. CT was performed as a low-dose CT scan without contrast enhancement during free breathing (100 kVp, 80 mAs/slice, collimation 20 × 1.25 mm, rotation time 0.66 s, pitch 1.5, matrix 512 × 512). SPECT, CT, and their fusion images were displayed in transverse, coronal, and sagittal planes. To ensure the same region of the body is represented in each of the numbered slices of every projection, it is preferable to register all three sets together. This approach facilitates the most dependable comparison between image sets for anatomical and functional correlation, ensuring the semi-automated volume of interest (VOI) of the localized lesion is both accurate and reproducible (Figure 1).

### 2.4. 99mTc-MIBI DPS Image Analysis

Abnormal hyperfunction of the parathyroid gland was considered to be focal areas of increased uptake with either delayed washout over time or fixed uptake that persisted on delayed imaging [13]. Lesions were localized according to six regions: lower left (*n* = 21), lower right (*n* = 16), upper left (*n* = 2), upper right (*n* = 7), intrathyroidal (lower middle right (*n* = 8), lower middle left (*n* = 5), upper middle right (*n* = 3), or ectopic (mediastinum or deep cervical; *n* = 8).

From the early and delayed planar DPS images, a region of interest (ROI) for the uptake lesion of the parathyroid adenoma was manually drawn using an image analysis tool. Another ROI of the same size and shape was reproduced in both the contralateral thyroid area and contralateral background cervical region (Figure 1A). The parathyroid adenoma/thyroid gland uptake ratio (LTR) was calculated as the ratio between the mean counts of the ROIs of the parathyroid adenoma and the contralateral thyroid gland [7]. Early LTR (eLTR) and late LTR (dLTR) were only used to calculate the retention index (RI) according to the following equation: RI = eLTR − dLTR/eLTR. The lesion/background uptake ratio (LBR) was calculated as the ratio between the mean counts of the ROIs of the parathyroid adenoma and the contralateral background cervical area. The early LBR (eLBR) and delayed LBR (dLBR) uptake ratios were expressed as radiopharmaceutical uptake of the adenomas.

The parathyroid adenoma volume (Svol) was measured as a SPECT-derived parameter via the volumetric SPECT option of the device (InterView^TM^ XP processing software, version 3.08.008.0000, Anyscan-SC, Mediso Ltd., Budapest, Hungary). A spherical or ellipsoid isocontour VOI was semi-automatically drawn around the focal uptake from the three orthogonal slices of reconstructed SPECT images with an image analysis tool for deducing the Svol (Figure 1B). A previously determined optimal threshold percentage of 40% was applied for parathyroid adenomas. Subsequently, the Parathormone/Svol (PTH/Svol) ratio was calculated.

To ensure the reproducibility and accuracy of the results, all planar and SPECT/CT parathyroid scans were retrospectively reviewed by two nuclear medicine specialists who were blinded to all clinical, radiological, and laboratory data. The final results were determined through consensus. In imaging measurements, the intraclass correlation coefficient for both intraobserver and interobserver variability ranged between 0.83 and 0.95 [14].

### 2.5. Statistical Analysis

Data, expressed as mean ± standard deviation (SD) or percentage were analyzed using SPSS software (version 22.0, IBM Corp., Armonk, NY, USA). Normally distributed data are presented as mean ± standard deviation (SD), non-normally distributed data as median (min–max), and categorical data as number (%). Independent *t*-test or Mann–Whitney U test was used to compare two independent groups, depending on the assumption of normality. Spearman’s rho correlation analysis was used for correlation analysis. ROC curve analysis was performed to determine the discriminative ability of the variables for disease severity and to determine the optimal cut-off values by the Youden index method [15]. Differences were considered statistically significant at *p*-values < 0.05.

## 3. Results

The characteristics of the patients and descriptive statistics for all variables are shown in Table 1. Twenty-eight patients underwent surgical procedures, followed by histopathological analysis.

**Figure 2:**
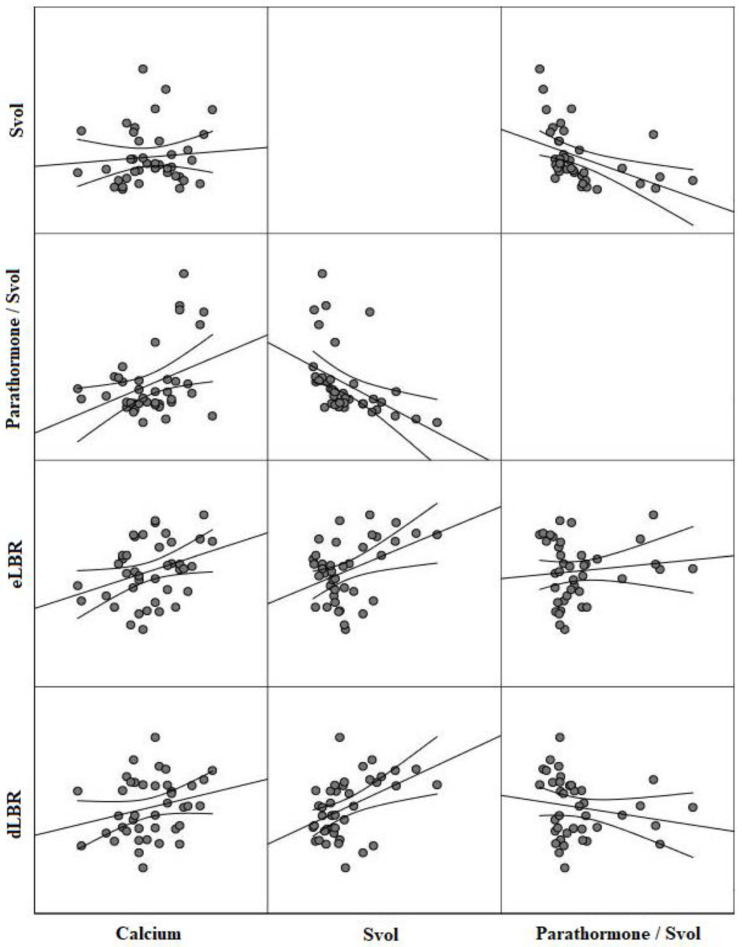
The correlation structure between calcium levels, Svol, PTH/Svol ratio, and radiopharmaceutical uptake.

PTH levels displayed a significant positive correlation with PTH/Svol levels (r = 0.59, *p* < 0.001). Calcium levels exhibited a positive correlation with radiopharmaceutical uptake parameters (r = 0.33, *p* < 0.001 for eLBR, r = 0.29, *p* = 0.01 for dLBR, and r = 0.31, *p* < 0.001 for dLTR) and PTH/Svol (r = 0.38, *p* < 0.001) (Figure 2). Calcium levels did not show a correlation with eLRT (r = 0.21, *p* = 0.28) and RI (r = 0.18, *p* = 0.23). Among the imaging parameters, only the PTH/Svol ratio showed a negative correlation with phosphorus levels (r = −0.29, *p* = 0.02). In addition, a positive correlation was found between radiopharmaceutical uptake parameters and Svol (r = 0.40, *p* < 0.001 for eLBR, r = 0.45, *p* < 0.001 for dLBR), but they were not correlated with PTH/Svol (Figure 2) (Table 2). There was a negative correlation between PTH/Svol and RI (r = −0.55, *p* < 0.001). However, there was no statistically significant correlation between functional markers and either Svol or RI (Table 2).

The median PTH, mean eLBR, median dLBR, median dLTR, and median PTH/Svol values were lower in mild hypercalcemia group compared to marked hypercalcemia group, while the median phosphorus levels were higher. The levels of Svol and RI did not show significant differences between the groups (Table 3).

**Figure 3:**
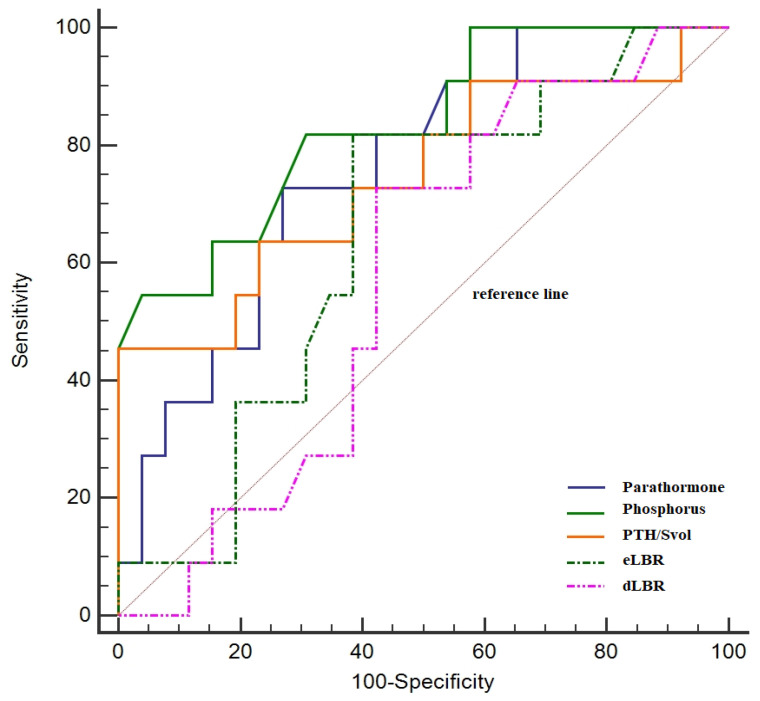
Diagnostic performance of functional markers and imaging parameters.

The ROC curve analysis demonstrated that PTH, phosphorus, eLBR, dLBR, dLTR, and the PTH/Svol ratio are significant markers in predicting the severity of the disease. Accordingly, PTH (68.42% sensitivity, 80.00% specificity), phosphorus (73.68% sensitivity, 73.91% specificity), and the PTH/Svol ratio (72.70% sensitivity, 62.10% specificity), which had different sensitivities and specificities, possessed similar AUC values (0.76, 95% CI = 0.64–0.85 for PTH vs. 0.75, 95% CI = 0.58–0.87 for phosphorus vs. 0.75, 95% CI 0.58–0.87 for PTH/Svol ratio; *p* > 0.05) (Figure 3). However, the AUC values of these parameters were higher than the AUC values of eLBR (0.68, 95% CI = 0.56–0.78), dLBR (0.66, 95% CI 0.54–0.77), and dLTR (0.66, 95% CI = 0.54–0.76). On the other hand, Youden index values were similar for PTH (0.48), Phosphorus (0.47), and eLBR (0.45), and were markedly higher than the PTH/SVOL ratio (0.35) (Table 4).

Most adenomas were located in the lower left, followed by the lower right. The mean serum calcium level was higher in adenomas localized on the lower right side when compared to those located on the lower left side (11.28 ± 0.72 vs. 10.74 ± 0.79 mg/dL, *p* = 0.05), while median phosphorus level was lower (2.40 vs. 3.10 mg/dL, *p* = 0.01). The mean eLBR level was higher in adenomas localized on the lower right side when compared to those located on the lower left side (2.76 ± 0.59 vs. 2.38 ± 0.47, *p* = 0.04) (Table 5). No relationship was detected between functional markers and imaging parameters and adenomas in other locations.

## 4. Discussion

This study stands as a pioneering effort, offering a comprehensive quantitative and volumetric scintigraphic analysis, elucidating the relationship between secretory capabilities and radiopharmaceutical uptake in the preoperative phase. Although previous research has investigated associations of adenoma magnitude and PTH measurements with the radiopharmaceutical uptake, the PTH/Svol has not been previously surveyed. In this research, a novel composite such as PTH/Svol was implemented for the first time, and examinations were conducted in accordance with disease severity. There was a significant relationship between functional parameters and both the quantitative and volumetric parameters of 99mTc-MIBI. Among the imaging parameters, PTH/Svol was associated with both the functional status of the parathyroid adenoma and the severity of the disease. Moreover, PTH/Svol exhibited a notable ability in forecasting marked hypercalcemia.

A meta-analysis demonstrated that the sensitivity of the 99mTc-MIBI DPS, SPECT/CT imaging for investigating solitary parathyroid adenoma, was ascertained to be 86%. This method is the most proficient for diagnosing functional parathyroid adenoma [16]. In addition to diagnosing a lesion, parathyroid imaging can be used for functional evaluation of a parathyroid adenoma, as 99mTc-MIBI binds to mitochondria based on the membrane potential [6,10,17]. Previous studies have reported a significant relationship between MIBI positivity and adenoma volume with calcium and PTH levels [6,7,8,9,10,18,19]. Radiopharmaceutical uptake significantly correlated with only serum calcium level, but functional markers were not correlated with Svol. These findings suggest that eLBR and dLBR might serve as imaging indicators, reflecting the effects of the parathyroid adenoma on calcium metabolism.

In MIBI scintigraphy, which is a functional imaging method, the volume of the adenoma can affect scan sensitivity. A prior study indicated that adenomas identified using MIBI scintigraphy were of a larger size compared to those that went undetected [20]. Conversely, several studies have indicated that the radiopharmaceuticals uptake was not significantly correlated with the size of the parathyroid adenoma [21,22]. On the other hand, it has been expressed that adenoma weight and serum PTH concentrations were also significant predictors for MIBI positivity [23,24]. Interesting findings revealed from a comprehensive study of patients with the uniglandular parathyroid disease are that a high PTH level, pathologic parathyroid gland volume, weight, and oxyphil cell content were all significantly associated with MIBI positivity [20]. The PTH per unit weight of the adenoma may be significantly lower in larger adenomas [25]. This hypothesis was confirmed by an in vitro study, which found that cells from larger glands released PTH at a lower rate than those from smaller adenomas [26]. Furthermore, larger adenomas may have a greater portion of nonfunctional spaces within the gland [25,27,28]. These factors may explain why there was no significant correlation between PTH/Svol and the radiopharmaceutical uptake in the current study. However, functional status of the parathyroid adenoma demonstrated a significant correlation with PTH/Svol, which suggests that the volume of metabolically active tissue may be a more effective marker than the simple metabolic volume. PTH/Svol is related to the functional load of the entire adenoma and is a novel measurable parameter that could possibly be composed of cross-sectional imaging and a functional hormonal marker.

While imaging parameters predicted disease severity with acceptable sensitivity, among these parameters, PTH/Svol demonstrated superior diagnostic performance. In clinical practice, it is critical to take note that the radiopharmaceutical uptake can indicate its size and capability, but not its sufficient PTH secretory potential. In a study using pathological volume, the PTH/volume ratio was found significantly lower in the MIBI positive group than in the MIBI negative group [29]. Therefore, it is noteworthy that this study found that the radiopharmaceutical uptake was positively related to the anticipated quantitative parameter Svol, but only the PTH/Svol parameter, which is a marker that may express the PTH secretory ability of an adenoma, was interlinked with all of the functional biochemical and hormonal characteristics. According to a comparative analysis of the ROC curve and Youden Index, quantitative analysis of PTH/Svol and eLBR showed similar diagnostic performance to PTH and phosphorus. Therefore, these parameters can be considered as innovative scintigraphic parameters that may be useful regarding patient management in clinical practice. The current study which encompassed the MIBI positive entire group also demonstrated that the ratio of PTH/Svol was lower in larger volume adenomas. Furthermore, as PTH/Svol was negatively correlated with RI, a low RI may point to a more productive adenoma, while a high RI may propose a less effective one. These findings also underscore the clinical importance of both early and delayed protocols [13]. There was a significant difference in eLBR, dLBR, and dLTR levels based on the severity of hypercalcemia. Furthermore, these parameters demonstrated superior diagnostic performance in predicting disease severity compared to Svol levels. Notably, the Youden index for dLTR was higher than that for eLTR. These findings suggest that delayed screening is necessary in PHPT cases.

An incremental value SPECT/CT over planar imaging for localization of abnormal parathyroid tissue in patients with primary hyperparathyroidism has been demonstrated in many studies [30,31]. In a meta-analysis study, the test performance of dual-phase 99mTc-sestamibi SPECT/CT in predicting the localization of parathyroid adenoma showed an estimated pooled sensitivity of 0.86 (95% CI = 0.81–0.90). SPECT/CT exhibited higher sensitivity compared to both SPECT (0.74; 95% CI = 0.66–0.82) and planar (0.70; 95% CI = 0.61–0.80) techniques [16]. In this study, no significant differences in volumetric parameters of SPECT imaging were observed between adenomas localized on the lower right and lower left sides. Additionally, adenomas localized in other regions had a limited sample size. Therefore, a comparison could not be made among all adenoma locations. On the other hand, in a recent study, it was reported that lower localized adenomas were larger in volume and weight than upper localized adenomas and that the detection ratio was higher for these reasons [32]. In comparing functional and imaging indicators in adenomas located on the lower right and lower left, the data of Ca, P, and eLBR indicate the lower right adenomas to be more functional.

This research has several limitations. It is a retrospective analysis in one clinic, and the likelihood of selection prejudice cannot be ruled out, despite the fact that the pictures were examined by readers unfamiliar with other clinical and pathological information. As this study was mainly based on scintigraphic volumetric measurements, only MIBI positive patients were included in the study and MIBI negative patients were not cared for. One limitation of this study is that it excluded the patients with MGD from its pool of participants. Only patients with single-gland disease were studied due to the desire to achieve clearer results in the volumetric study. Additionally, due to the fact that not all participants had pathology results available, it was not possible to compare imaging parameters with pathologic cellular type, adenoma volume, and adenoma weight. Moreover, the current study’s findings might be less conclusive due to the limited patient sample size. Therefore, larger prospective studies are needed to ascertain the effectiveness of the volumetric analysis method in evaluating parathyroid adenoma using SPECT/CT.

## 5. Conclusions

This study indicates a positive correlation between calcium levels and certain imaging parameters (eLBR, dLBR, dLTR, and PTH/Svol). However, the PTH/Svol ratio was the only imaging parameter that showed a relationship with phosphorus levels. Despite being an older parameter, the RI lacked an association with functional markers and inversely correlated with PTH/Svol. Therefore, the combined use of early and delayed planar 99mTc-MIBI DPS and SPECT/CT scans can provide valuable insights into both the functional status and disease severity of a parathyroid adenoma and may serve as more reliable indicators.

## Figures and Tables

**Table 1:** Demographic and clinical findings of PHPT patients.

Variables	Study Population *n* = 70
Demographic findings	
Age, year	56.23 ± 13.14
Gender, *n* (%)	
Male	16 (22.90)
Female	54 (77.10)
Severity of hypercalcemia, *n* (%)	
Mild	50 (71.40)
Severe	20 (28.60)
Laboratory findings	
Parathormone, ng/L	128.30 (33.10–888.00)
Calcium, mg/dL	11.02 ± 0.71
Phosphorus, mg/dL	2.71 (1.61–5.30)
25 (OH) vitamin D, µg/L	14.20 (4.20–52.20)
Localization of adenoma, *n* (%)	
Lower left	21 (30.00)
Lower right	16 (22.90)
Lower middle right	8 (11.40)
Upper right	7 (10.00)
Lower middle left	5 (7.10)
Upper middle right	3 (4.30)
Upper left	2 (2.90)
Ectopic	8 (11.40)
Planar imaging parameters	
eLBR	2.61 ± 0.67
eLTR	1.33 ± 0.32
dLBR	2.06 (0.22–4.01)
dLTR	1.28 (0.13–2.32)
RI	0.03 [(−0.90)–0.96]
SPECT imaging parameters	*n* = 41
Svol, cm^3^	2.10 (0.64–8.23)
PTH/Svol ratio, ng/L/cm^3^	55.06 (6.97–288.33)

Data are mean ± standard deviation or median (min–max), or number (%). eLBR, early lesion to background ratio; eLTR, early lesion to thyroid ratio; dLBR, delayed lesion to background ratio; dLTR, delayed lesion to thyroid ratio; PTH/Svol ratio, parathormone to scintigraphic volume ratio; RI, retention index; Svol, scintigraphic volume.

**Table 2:** Correlations between functional markers and imaging parameters.

Variables	Svol ^†^	PTH/Svol Ratio ^†^	eLBR	dLBR	RI
Parathormone					
r	0.20	0.59	0.09	0.08	0.06
* p*-value	0.25	<0.001	0.66	0.79	0.80
Calcium					
r	0.12	0.38	0.33	0.29	0.18
* p*-value	0.46	<0.001	<0.001	0.01	0.23
Phosphorus					
r	−0.08	−0.29	−0.15	−0.20	−0.05
* p*-value	0.62	0.02	0.29	0.15	0.87
25 (OH) vitamin D					
r	0.12	−0.17	−0.11	−0.05	0.02
* p*-value	0.46	0.32	0.32	0.87	0.99
eLBR					
r	0.40	−0.07	-	0.70	−0.14
* p*-value	<0.001	0.69	-	<0.001	0.26
eLTR					
r	−0.04	0.21	0.60	0.38	−0.43
* p*-value	0.79	0.20	<0.001	0.01	<0.001
dLBR					
r	0.45	−0.19	0.70	-	0.20
* p*-value	<0.001	0.30	<0.001	-	0.17
dLTR					
r	0.28	−0.10	0.49	0.49	0.62
* p*-value	0.08	0.54	<0.001	<0.001	<0.001
RI					
r	0.61	−0.55	−0.14	0.20	-
* p*-value	<0.001	<0.001	0.26	0.17	-
Svol ^†^					
r	-	−0.66	0.40	0.45	0.61
* p*-value	-	<0.001	<0.001	<0.001	<0.001

^†^ It was analyzed in 41 patients. eLBR, early lesion to background ratio; eLTR, early lesion to thyroid ratio; dLBR, delayed lesion to background ratio; dLTR, delayed lesion to thyroid ratio; PTH/Svol ratio, parathormone to scintigraphic volume ratio; RI, retention index; Svol, scintigraphic volume.

**Table 3:** Comparison of functional markers and imaging parameters based on the severity of hypercalcemia.

Variables	Severity of Hypercalcemia		*p*-Value
	Mild	Marked	
	(10.50–11.49 mg/dL)	(>11.50 mg/dL)	
	*n* = 50	*n* = 20	
Parathormone, ng/L	119.50 (33.10–374.00)	172.00 (74.80–888.00)	0.002
Phosphorus, mg/dL	2.85 (1.98–4.83)	2.32 (1.61–5.30)	0.001
25 (OH) vitamin D, µg/mL	14.75 (4.20–52.20)	11.90 (6.72–37.40)	0.471
Planar imaging parameters			
eLBR	2.51 ± 0.64	2.91 ± 0.63	0.020
eLTR	1.29 ± 0.33	1.42 ± 0.32	0.092
dLBR	1.92 (0.22–4.01)	2.28 (1.65–3.68)	0.038
dLTR	1.24 (0.13–1.87)	1.43 (0.89–2.32)	0.010
RI	0.01 [(−0.90)–0.96]	0.07 [(−0.14)–0.60]	0.132
SPECT imaging parameters	*n* = 29	*n* = 12	
Svol, cm^3^	2.20 (0.64–8.23)	2.27 (0.70–5.67)	0.682
PTH/Svol ratio, ng/L/cm^3^	45.44 (6.97–158.60)	84.56 (19.22–288.33)	0.017

Data are mean ± standard deviation or median (min–max). eLBR, early lesion to background ratio; eLTR, early lesion to thyroid ratio; dLBR, delayed lesion to background ratio; dLTR, delayed lesion to thyroid ratio; PTH/Svol ratio, parathormone to scintigraphic volume ratio; RI, retention index; Svol, scintigraphic volume.

**Table 4:** Cut-off values, sensitivity, and specificity of the variables in the prediction of severe disease.

Variables	AUC (95% CI)	Cut-Off Value	*p*-Value	Sensitivity (%)	Specificity (%)	Youden index
Parathormone	0.76 (0.64–0.85)	>150.00	<0.001	68.42	80.00	0.48
Phosphorus	0.75 (0.58–0.87)	<2.53	<0.001	73.68	73.91	0.47
25 (OH) vitamin D	0.56 (0.43–0.68)	<15.57	0.22	76.47	45.67	0.22
Planar imaging parameters						
eLBR	0.68 (0.56–0.78)	>2.54	0.01	85.00	60.00	0.45
eLTR	0.62 (0.49–0.73)	>1.45	0.13	45.80	79.17	0.25
dLBR	0.66 (0.54–0.77)	>2.13	0.03	65.00	66.00	0.31
dLTR	0.66 (0.54–0.76)	>1.40	0.01	60.00	75.00	0.35
RI	0.62 (0.49–0.73)	>0.07	0.11	50.00	68.80	0.19
SPECT imaging parameters						
Svol	0.54 (0.38–0.70)	<1.50	0.68	41.67	79.31	0.21
PTH/Svol ratio	0.75 (0.58–0.87)	>58.35	0.01	72.70	62.10	0.35

eLBR, early lesion to background ratio; eLTR, early lesion to thyroid ratio; dLBR, delayed lesion to background ratio; dLTR, delayed lesion to thyroid ratio; PTH/Svol ratio, parathormone to scintigraphic volume ratio; RI, retention index; Svol, scintigraphic volume.

**Table 5:** Comparisons between groups according to adenoma localization.

Variables	Anatomic Localization		*p*-Value
	Lower Right *n* = 16	Lower Left *n* = 21	
Parathormone, ng/L	126.00 (67.10–888.00)	135.00 (33.10–346.00)	0.28
Calcium, mg/dL	11.28 ± 0.72	10.74 ± 0.79	0.05
Phosphorus, mg/dL	2.40 (2.10–3.60)	3.10 (1.93–4.83)	0.01
25 (OH) vitamin D, µg/mL	12.15 (9.52–37.40)	16.20 (4.20–52.20)	0.58
Planar imaging parameters			
eLBR	2.76 ± 0.59	2.38 ± 0.47	0.04
eLTR	1.46 ± 0.28	1.30 ± 0.37	0.14
dLBR	2.01 (0.22–3.46)	1.90 (1.56–3.30)	0.34
dLTR	1.34 (0.13–2.17)	1.20 (0.88–2.30)	0.08
RI	0.01 [(−0.90)–0.24]	0.02 [(−0.40)–0.60]	0.55
SPECT imaging parameters			
SVOL, cm^3^	1.50 (0.64–3.70)	1.84 (1.00–4.54)	0.36
PTH/Svol ratio, ng/L/cm^3^	84.22 (35.14–227.97)	62.10 (31.06–288.33)	0.42

Data are mean ± standard deviation or median (min–max). eLBR, early lesion to background ratio; eLTR, early lesion to thyroid ratio; dLBR, delayed lesion to background ratio; dLTR, delayed lesion to thyroid ratio; PTH/Svol ratio, parathormone to scintigraphic volume ratio; RI, retention index; Svol, scintigraphic volume.

## Data Availability

The data that support the findings of this study are available on request from the corresponding author.
